# Supplementary material for: Effects of C-Terminal Lys-Arg Residue of AapA1 Protein on Toxicity and Structural Mechanism
Source: Toxins (Basel). 2023 Sep 2;15(9):542. doi: 10.3390/toxins15090542 (PMC10537873; doi:10.3390/toxins15090542)
Supplement: Supplementary file 1 [file toxins-15-00542-s001.zip › toxins-2564962-supplementary.pdf]

# Supplementary Materials: Effects of C Terminal Lys Arg Residue of AapA1 Protein on Toxicity and Structural Mechanism

**Table S1. Number of contacts and binding free energy (kJ/mol) between AapA1-28 and the POPE/POPG (3:1) lipid bilayer during the last 200 ns trajectories in CMD simulations**

| | Contacts number | ΔGbind | ΔEele | ΔEvdw | ΔGpol | ΔGnonpol |
|---|---|---|---|---|---|---|
| CMD_M1 | 12399 799 | -4031±203 | -5226±320 | -715±54 | 2003±278 | -93±6 |
| CMD_M2 | 13926 823 | -4161±141 | -5460±219 | -842±50 | 2222±207 | -81±4 |
| CMD_M3 | 13478 740 | -3907±199 | -4911±378 | -817±60 | 1924±294 | -104±6 |
| CMD_M4 | 13600 775 | -4272±211 | -5405±293 | -802±66 | 2015±286 | -80±6 |
| CMD_M5 | 11283 729 | -3800±180 | -4572±332 | -681±45 | 1539±313 | -87±5 |

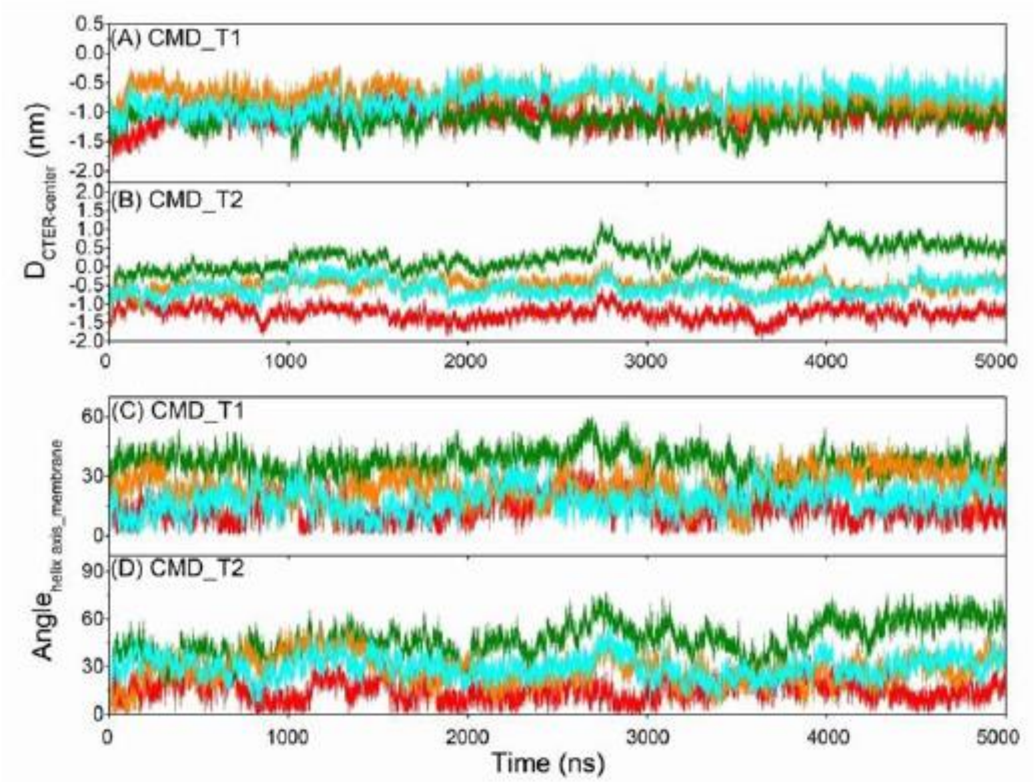


**Figure S1. The time series of distance between C-terminal five residues and membrane center and angle between protein helical axis and membrane Z-axis in simulations CMD_T1 and CMD_T2. Red line: the first AapA1-28 protein; green line: the second AapA1-28 protein; orange line: the third AapA1-28 protein; cyan line: the fourth AapA1-28 protein.**

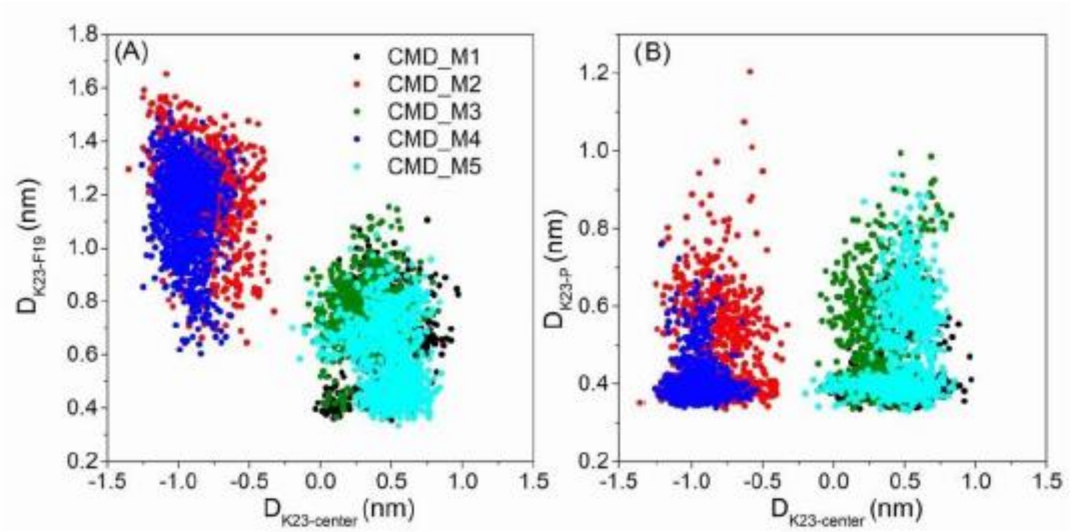


**Figure S2. Scatter plot of the relationship of (A) distance between K-23 sidechain and membrane center vs distance between K-23 ammonium NH3+ and F-19 aromatic ring, and (B) distance between K-23 sidechain and membrane center vs distance between K-23 ammonium NH3+ and the closest phosphate group of membrane in simulation CMD_M.**
